# Expected a posteriori scoring in PROMIS^®^

**DOI:** 10.1186/s41687-022-00464-9

**Published:** 2022-06-03

**Authors:** Robert Chapman

**Affiliations:** Department of Medical Social Sciences, Feinberg School of Medicine, Northwestern University, 625 North Michigan Avenue, Chicago, IL 60601 USA

## Abstract

**Background:**

The Patient-Reported Outcome Measurement Information System^®^ (PROMIS^®^) was developed to reliably measure health-related quality of life using the patient’s voice. To achieve these aims, PROMIS utilized Item Response Theory methods in its development, validation and implementation. PROMIS measures are typically scored using a specific method to calculate scores, called Expected A Posteriori estimation.

**Body:**

Expected A Posteriori scoring methods are flexible, produce accurate scores and can be efficiently calculated by statistical software. This work seeks to make Expected A Posteriori scoring methods transparent and accessible to a larger audience through description, graphical demonstration and examples. Further applications and practical considerations of Expected A Posteriori scoring are presented and discussed. All materials used in this paper are made available through the R Markdown reproducibility framework and are intended to be reviewed and reused. Commented statistical code for the calculation of Expected A Posteriori scores is included.

**Conclusion:**

This work seeks to provide the reader with a summary and visualization of the operation of Expected A Posteriori scoring, as implemented in PROMIS. As PROMIS is increasingly adopted and implemented, this work will provide a basis for making psychometric methods more accessible to the PROMIS user base.

## Introduction

The Patient-Reported Outcome Measurement Information System^®^ (PROMIS^®^) [1], is a disease-agnostic measurement system of health-related quality of life which utilizes Item Response Theory (IRT). PROMIS was originally created to leverage the benefits of IRT and Computer Adaptive Testing (CAT) to minimize patient response burden while maximizing measurement reliability. PROMIS measures have been shown to be reliable, valid and accurate in a variety of conditions and contexts [2–7]. Over the past fifteen years, there has been substantial development, adoption and implementation of PROMIS [8, 9]. Such efforts have leveraged IRT to increase the accessibility of and aid their interpretation, including T-score maps [10] and “linking” between non-PROMIS and PROMIS measures [11].

This paper aims to make PROMIS IRT scoring methods accessible to a broader audience of users who have a basic statistical background by supplementing foundational psychometric literature with non-technical descriptions and illustrative graphics. To the same end, this paper was created in the reproducibility framework of R Markdown [12]. An R Markdown document (.rmd) contains both commented statistical code and the explanatory text in this document. Both the text and statistical code for scoring is intended to be reviewed and implemented by the reader. Included in the appendices of this paper are a set of annotated statistical programming scripts for scoring PROMIS measures.

## IRT foundations

The IRT methods employed in PROMIS and their foundations were developed 70–90 years ago [13–17] and have been used extensively in the educational field. Over the past two decades, researchers have also shown how IRT can be applied to patient-centered outcomes generally [18, 19] and documented how IRT has been applied in PROMIS specifically [8, 9]. This paper briefly reviews foundations of IRT in PROMIS and instead provides focused demonstration of PROMIS scoring methods.

## Response option probabilities

### Building block of IRT scoring

IRT ranks individuals and their responses to survey items across a latent trait, such as fatigue. Just as two different people might have different levels of fatigue, two different sets of responses to survey items relate to two different levels of fatigue. IRT allows us to infer where an individual most likely ranks on a latent trait continuum. The inference of where an individual ranks on a latent trait is made by transforming an individual’s response to survey items (e.g., *I feel tired*—*Never*, *Sometimes*, and *Always*) to a set of probabilities across all levels of the latent trait. Each probability in the set represents the likelihood that an individual and their selected response options has a particular level of latent trait. Expected A Posteriori scoring reduces these probability sets to a single point-estimate of the latent trait (i.e., a score) and provides an estimate of variability and reliability of the point estimate of the latent trait (i.e., standard deviation or standard error).

Two things are required to calculate these probabilities for a PROMIS measure: item calibration parameters, such as those shown in Table 1, and the two parameter logistic IRT model shown in Formula (1). The calibration parameters represent the relationship between a sample of individuals, their responses to a set of survey items and the latent trait. The formula allows a mathematical transformation of an individual’s response to an item to a set of probabilities across the spectrum of the latent trait.

**Table 1:** IRT Calibration Statistics for PROMIS Fatigue item FATEXP42: *How much mental energy did you have on average?*

a	cb1	cb2	cb3	cb4
1.44	− 1.26	0.78	1.95	3.51

In Formula (1) we can see the calibration parameters, annotated as “discrimination” and “threshold.” Each item has one discrimination calibration parameter and a number of threshold calibration parameters equal to the number of response options minus one. The subscript “i” in Formula (1) indicates that these parameters vary by item, and the subscript “k” indicates that there are multiple thresholds per item. An example is PROMIS Fatigue item FATEXP42 (*In the past 7 days, how much mental energy did you have on average?*) which has five response options (*Not at all*, *A little bit*, *Somewhat*, *Quite a bit*, and *Very much*). It follows that FATEXP42 has one discrimination calibration parameter (abbreviated “a”), and four threshold calibration parameters (abbreviated and numbered from “cb1” to “cb4”). The item calibrations parameters for FATEXP42 are provided here in Table 1 for reference.

The remaining undefined variable in Formula (1) is “theta,” which refers to the latent trait being measured (e.g., fatigue or physical functioning). Theta is actualized as a single number for an individual level of latent trait, ranging from negative infinity to infinity. Theta is constructed based on the population included in the calibration sample and is often scaled to have a mean center of 0 and a standard deviation of 1. For the PROMIS Profile measures (that include anxiety, depression, fatigue, pain, sleep disturbance, physical function, and satisfaction with participation in social roles), Cella and Liu [1, 20] provide a picture of the people representing the PROMIS calibrations and metric. An individual’s theta score represents their level of latent trait in the context of the sample that was used to generate the calibration parameters.

For the purposes of calculation, the range of theta is limited to ± 4, with a higher theta relating to more of what is being measured, e.g., higher PROMIS Fatigue theta values relate to more fatigue or higher PROMIS Physical Function theta values relate to better physical functioning.

1 $$Probability = \frac{1}{{1 + e^{{ - 1{*}discrimination_{i} \left( {theta - threshold_{ik} } \right)}} }}$$

**Fig. 1:**
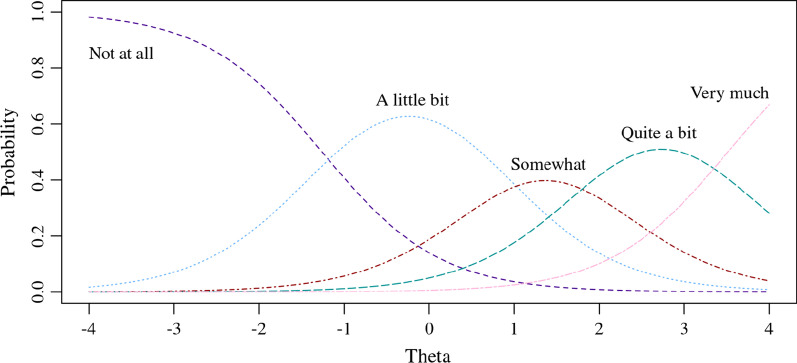
Response option probabilities across theta for PROMIS Fatigue item FATEXP42

Once we evaluate Formula (1) for all levels of theta (e.g., − 4 to 4) and for all item calibrations parameters provided in Table 1, we can create a set of probability curves that represent each item’s response options. Figure 1 shows an example of how the response options of FATEXP42 are ordered across level of theta (level of fatigue), with response option *Not at all* having higher probabilities at lower levels of theta (lower fatigue), and response option *Very much* having higher probabilities at higher levels of theta (higher fatigue).

This paper demonstrates how PROMIS measures are scored using graphical representations of probability curves, such as those in Fig. 1. To aid interpretation, these probability curves are plotted with consistent formatting styles. All colors used in figures were selected from the *colorBlindness* package in R [21].

**Fig. 2:**
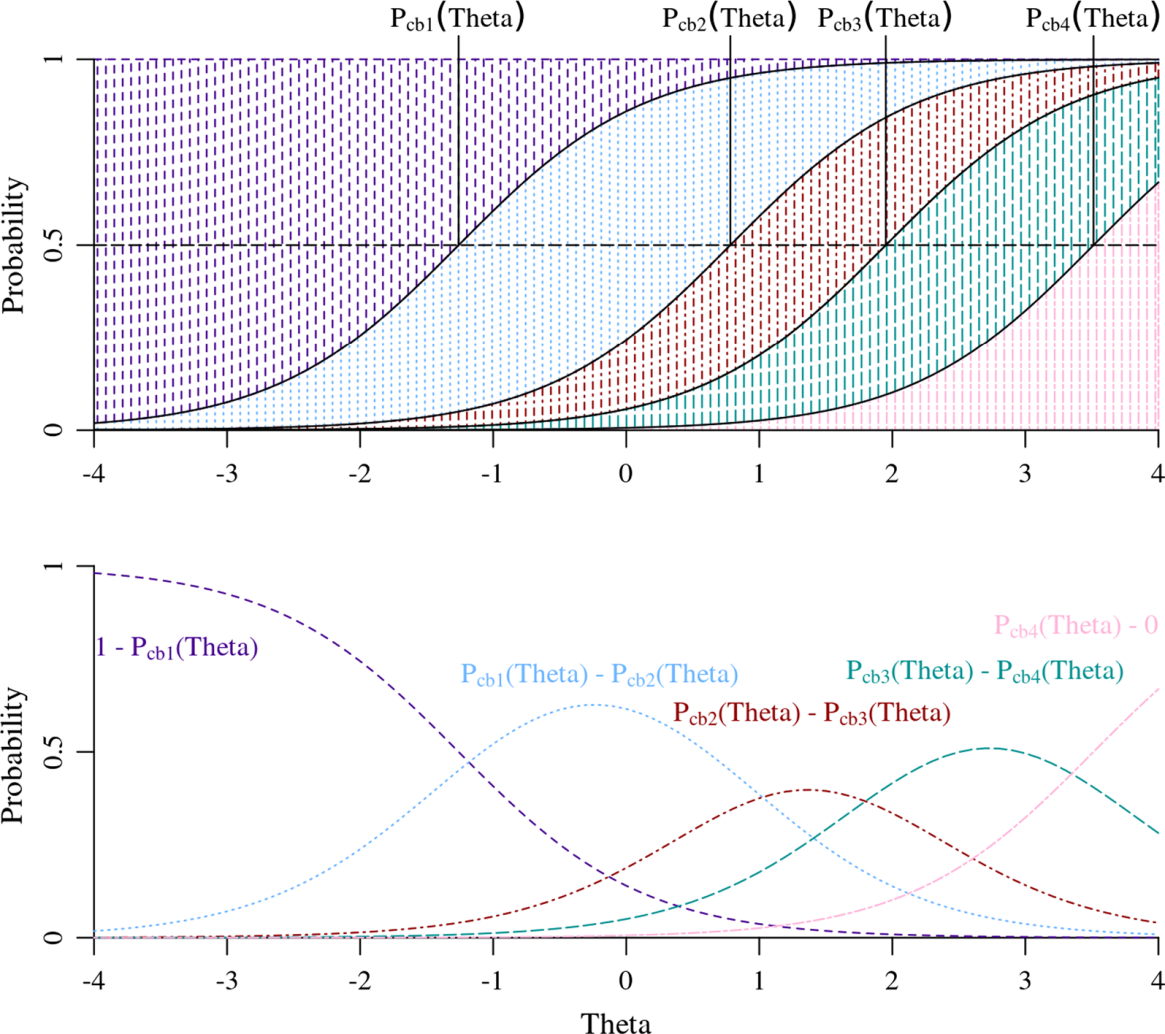
Calculation of response option probabilities across theta using the graded response model calibrations from PROMIS Fatigue item FATEXP42

Figure 2 provides a more detailed example of how Formula (1) and FATEXP42’s item calibration parameters can be used to generate sets of probabilities and plot what are referred to as item characteristic curves. The black curves in the top graph of Fig. 2 are calculated with Formula (1) and the calibration parameters in Table 1 and are labeled as the “probability associated with a threshold parameter across theta” or P_cb1−cb4_(Theta). These curves represent the probability that a respondent at a given level of theta would endorse any response option above one of the response options, e.g., P_cb2_(Theta) represents the probability that an individual would endorse the third, fourth or fifth response option (*Somewhat*, *Quite a bit*, and *Very much*), but not the first or second response option (*Not at all* and *A little bit*). The threshold parameters (e.g., cb1 = − 1.26) represents value of theta where its corresponding threshold probability curve reaches 0.5, as represented by the intersection of the dotted black horizontal line and the vertical line segments underneath the threshold probability curve labels.

The bottom plot of Fig. 2 presents the same item characteristic curves in Fig. 1, but with the response option probability curves labeled with their calculations. To isolate the probability associated with an individual response option, we calculate a set of probability differences between the probability curves of adjacent thresholds [e.g., P_cb2_(Theta)—P_cb3_(Theta)]. The last threshold probability curve, P_cb4_(Theta), does not have an adjacent threshold probability because the item FATEXP42 does not have a response option greater than the fifth (*Very Much*). To calculate the probability associated with the fifth and highest response option, we subtract P_cb4_(Theta) from 0. In other words, the probability associated with a respondent endorsing the fifth and highest response option is equal to the probability that a respondent will endorse any response option above the fourth, P_cb4_(Theta), minus the impossibility (0 probability) that a participant will endorse a response option higher than fifth and highest. The first threshold probability curve, P_cb1_(Theta), does not have another threshold probability curve below it. To calculate the probability associated with the lowest response option (*Not at all*) we subtract P_cb2_(Theta) from 1. In other words, the probability of respondent endorsing the lowest response option is equal the certainty (1 probability) that a participant will endorse any response option minus the probability that a respondent will endorse a response option above the first and lowest, P_cb1_(Theta).

The procedure of subtracting adjacent threshold probability curves to obtain probabilities curves of individual response options is reflected in the graded response model, Formula (2). To generate probabilities, we find the difference between two equations, one with threshold “k” and the other with threshold “k + 1”. The graded response model formula is the companion equation for interpreting PROMIS item calibration statistics and calculating probabilities. Although originally published by Samejima, the graded response model is explained in more accessible terms by Reeve, Chang, Fayers and Embretson [13, 19, 22, 23].

2 $$Probability = \frac{1}{{1 + e^{{ - 1{*}a_{i} \left( {theta - cb_{ik} } \right)}} }} - \frac{1}{{1 + e^{{ - 1{*}a_{i} \left( {theta - cb_{ik + 1} } \right)}} }}$$

## Expected a posteriori scoring

### How do we go from IRT probabilities to scores?

IRT provides probability-based modeling to evaluate item- and scale-level characteristics for scale development, but we can also use IRT to find an estimate of where an individual is on the theta spectrum. In other words, we can score individuals on the latent trait. PROMIS scores are reported on the “T-score” metric, which is a linear transformation of the standardized theta scores, as shown in Formula (3). This paper reports scores on either the standardized z-score metric (labeled "theta") or the T-score metric.

3 $$T - score = \left( {theta{*}10} \right) + 50$$

**Fig. 3:**
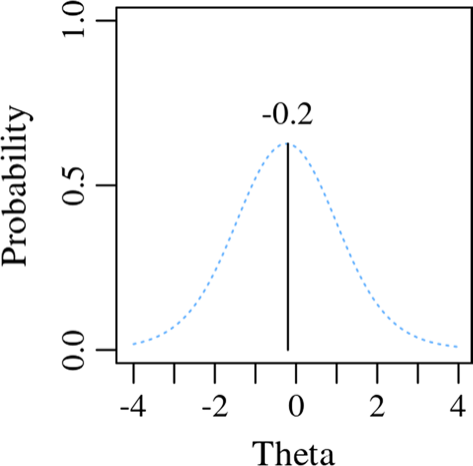
*A little bit* response option probabilities across theta for PROMIS Fatigue item FATEXP42

As a score calculation example, we will again use PROMIS Fatigue item FATEXP42 (*In the past 7 days, how much mental energy did you have on average?*). See item response option probability curve for the second response option (*A little bit*) in Fig. 3. A logical IRT score is the most probable level of theta, also known as the maximum likelihood of theta. Using this method, an individual that selected the second response option of FATEXP42 would be assigned a maximum likelihood score of − 0.2 theta or T-score of 48, as shown in Fig. 3.

This simple example has two problems, however. The first problem comes from a practical issue in measurement and the second stems from mathematical limitations. The practical measurement issue is that we are unable to differentiate individuals at the extreme ends of our measurement scale, which occurs when respondents select the absolute highest or lowest response option in an item (e.g., *Never* or *Always*). Using another fatigue item as an example, in FATEXP29 (*In the past 7 days, how often did you feel totally drained?*) the extreme response of *Never* is likely selected by people with very different experiences of fatigue: *Never* would be selected a by respondent with low-level fatigue (e.g., feels slightly, but not totally drained over the past week), *Never* would be selected by a respondent who didn’t experience fatigue (e.g., didn’t feel drained at all over the past week) and *Never* would be selected by a respondent who had an unusually high energy over the past week. While the extreme response option of *Never* is selected by all three respondents for this item, we can be more certain that respondents with even less fatigue (or more energy) are increasingly likely to pick the *Never* response option.

**Fig. 4:**
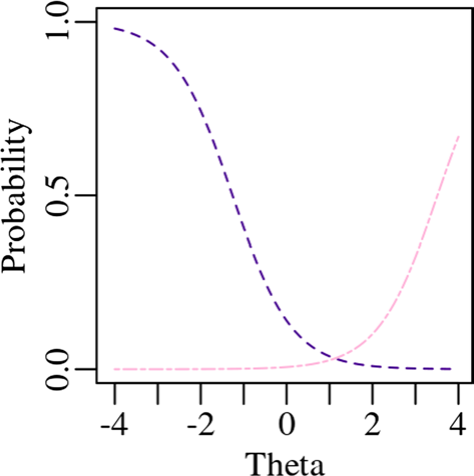
Extreme response option probabilities across theta for PROMIS Fatigue item FATEXP42

This is also true for the other extreme response option, *Always*. A response of *Always* is likely to be selected by a respondent who just had a totally draining week, by a respondent who had a totally draining month, or by a respondent who had a totally draining year. The inability of an item or scale to distinguish between extreme levels is a measurement property known as the “floor” and “ceiling” effect [24]. The response probability curves of the extreme responses options show these floor and ceiling effects (Fig. 4). Probabilities of these extreme responses categories are assumed to be monotonic, meaning they have a constantly increasing probability of being selected with increasingly extreme levels of theta, and there is no single point of maximum likelihood for us to use as a score.

The second problem related to mathematical limitations is the infinite range that we assume exists for the latent trait (theta). All response option probability curves are asymptotic, meaning the probability curves expand over an infinite range of theta and never reach probability values of either 0 or 1. It is mathematically complex and computationally costly to perform calculations in an infinite range.

**Fig. 5:**
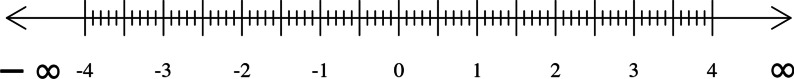
Theta quadrature as number line

To solve these two problems, we use an IRT scoring mechanism called “Expected A Posteriori” (EAP) scoring [15, 25]. This form of scoring works by imposing constraints on how we calculate probabilities. The first constraint comes from limiting the infinite theta space to a “quadrature,” which can be visualized in Fig. 5 as a set of evenly spaced points on a number line or x axis between two bounds. Boundaries of − 4 theta to 4 theta or (T-scores of 10–90), with 0.1 theta increments (1 T-score point) are used. Theta can be interpreted as standard deviations of the population, a range of − 4 to 4 theta encompasses 99.994% of people.

The quadrature stops the constant growth of the extreme response option’s probability curve at its limits ( − 4 to 4), which means that a ‘maximum likelihood’ theta score for an extreme response option will be the same as the quadrature limit. Expanding or shrinking the limits of the quadrature (e.g., − 6 to 6 or − 2 to 2) will increase or decrease the scores of extreme response options. An individual who endorses an extreme response option would receive different theta scores only due to the choice of quadrature limits, not any real difference in the latent trait (e.g., fatigue).

EAP scoring uses a “prior” in the calculation of scores to address this problem. Generally, a prior is a bayesian concept that refers to our best guess of an individual’s theta score before they’ve selected a response option [24]. The EAP scoring prior used in PROMIS is a normal distribution which reflects the population mean (μ = 0) and standard deviation (σ = 1). It is a reasonable assumption that any individual is a member of the population.

After multiplication of the item characteristic curve by the normal prior probability curve, the extreme response probability curve is reshaped, repositioned and called the “posterior probability.” The new posterior probability curve is pulled back from the quadrature limit and is no longer monotonic: instead it looks like the normal curve of the prior. The amount of the lateral repositioning of the posterior (and movement of the maximum likelihood score) away from the quadrature limit is a function of the area under the curve of the original extreme response option probability and the area under the curve of the prior.

**Fig. 6:**
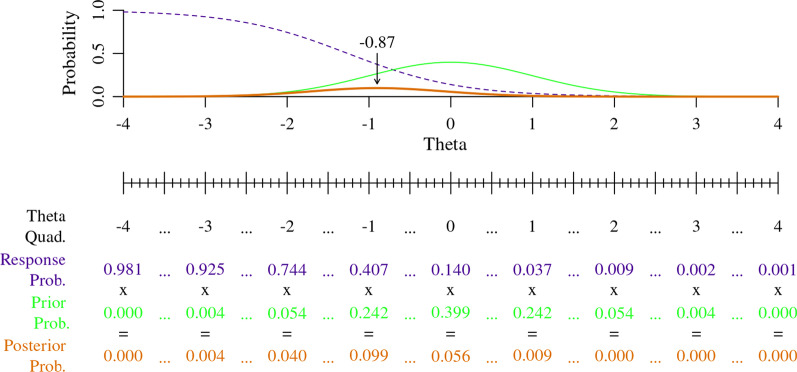
Calculation of extreme response option probabilities across theta for PROMIS Fatigue item FATEXP42

Figure 6 shows a graphical example of the new posterior curve. In Fig. 6, the dashed purple line represents the response probabilities from FATEXP42’s extreme response option (*Not at all*), the solid green line represents the prior probability curve, and the bold solid orange line represents the new posterior probability curve with a maximum probability of − 0.87. The posterior (bold solid orange) can be visualized as ‘splitting the difference’ between the probability curves of the extreme response option (dashed purple) and the prior (solid green).

The bottom half of Fig. 6 shows the calculation of the posterior probability curve using the theta quadrature. At each increment on the theta quadrature (− 4 to 4 by increments of 0.1), the response option probability is multiplied by the prior probability. For example, at a theta of − 1, the response probability of 0.407 is multiplied by a prior probability of 0.242, which equals a posterior probability of 0.099. The size of the posterior probabilities are shrunk due to the multiplication of decimals, but we are only concerned with the location of the maximum likelihood point estimate that we’ll use as an EAP score. Without the theta quadrature, integral calculus would be required to multiply the prior and the response option probability curves.

**Fig. 7:**
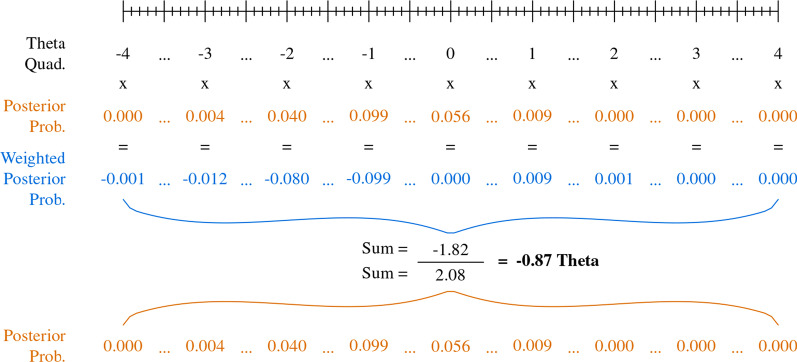
Calculation of theta for PROMIS Fatigue item FATEXP42, *Not at all* response option only

Figure 7 further demonstrates the method for calculating a single theta score from posterior probabilities across the theta quadrature. The quadrature again allows us to use simple multiplication in lieu of calculus, by multiplying posterior probabilities at each theta increment by their corresponding theta level to create a set of theta weighted posterior probabilities, e.g., theta of − 2 multiplied by a posterior probability of 0.04 equals a weighted probability of − 0.08. Dividing the sum of the weighted posterior probabilities ( − 1.82) by the sum of the posterior probabilities (2.08) gives us the final theta estimate ( − 0.87).

We originally introduced the prior into the scoring calculation in order to circumvent problems with extreme responses. However, in order to make sure that scores from all response options (extreme or not) are comparable, the prior is used in calculating all scores. This is also true for scores calculated from multiple items.

To calculate a single score from an individuals’ responses to multiple items, we combine the probability curves through multiplication. This operation is analogous to calculating the joint probability of two independent events, e.g., the probability of obtaining two heads from two coin flips is calculated as 0.5 × 0.5 = 0.25. A combined probability can then be multiplied by the prior to obtain a posterior probability.

**Fig. 8:**
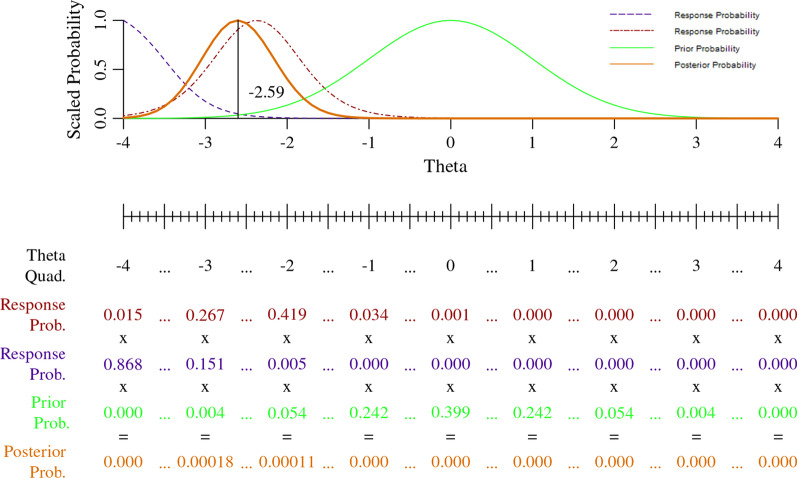
Expected A Posteriori scoring with multiple PROMIS Physical Function items

In calculating a score from multiple items, we multiply all response probabilities together, and then multiply by the prior to generate a set of single set of posterior probabilities, as in Fig. 8 below. Figure 8 uses two response options probabilities from PROMIS Physical Function items PFA56 (*Are you able to get in and out of a car?*) and PFC46 (*Are you able to transfer from a bed to a chair and back?*). The calibration statistics for PFA56 and other PROMIS Physical Function items mentioned in this work can be found in the first Table of Rose et al. [26] without the “PF” item code prefix, e.g., “A56” is the same as “PFA56.”

The probabilities in the graph of Fig. 8 are scaled to make the posterior probability curve more visible. The dashed purple line represents the scaled response probabilities for extreme response option of PFC46, *Unable to do* and the dot-dashed brown line represents the scaled response probabilities of PFA56, *With some difficulty*. The solid green line represents the scaled prior probabilities and bold solid orange line represents the scaled probabilities of the posterior. The process for calculating a single theta score from multiple items is the same as in the single item example in Fig. 7.

### Practical considerations of EAP scoring

There are three practical considerations of EAP scoring: one consideration related to the ordering of items, one related to score resolution, and another related the bias of prior.

Figure 8 shows that simple multiplication can be used to combine IRT response probabilities of multiple items. A property of multiplication is that any order or arrangement of multiplications has the same result (e.g., 1 × 2 × 3 = 3 × 2 × 1). Consequently, the order of items doesn’t matter in score calculation; item responses combined in any order will result in the same score.

The insensitivity to item order in IRT scoring also means that the resolution of scores increases exponentially with the number of items answered. One item with five response options has 5 possible IRT scores (5^1^ = 5), two items have 25 possible IRT scores (5^2^ = 25) and three items have 125 possible IRT scores (5^3^ = 125). This is a large increase in score resolution over raw sum scoring methods, in which the same three items have only 13 possible sum scores, ranging from 3 to 15. Greater score resolution allows scores to be more sensitive to an individual’s responses to a set of items and is a component of score precision.

As shown in Fig. 6, multiplication by the prior biases an EAP score inward. However, since the prior is only multiplied once in calculation of the posterior, it’s influence on the final EAP score will shrink as more items added into the calculation.

For these reasons, this paper doesn’t recommend EAP scoring with fewer than 3 items. The shortest PROMIS Profile short form has 4 items and adult PROMIS CAT will administer 4 items as the standard minimum. There are few PROMIS short forms with less than 4 items, including 2 item Global Physical and Mental Health scales [27].

### Raw sum score to IRT look-up table scoring

The previous sections demonstrate that Expected A Posteriori scoring is flexible and can be efficiently calculated by computers, but requires both statistical coding and calibration parameters to generate scores from item responses. For PROMIS users who do not have access to statistical code or calibration parameters, the HealthMeasures Scoring Service (https://www.assessmentcenter.net/ac_scoringservice) allows users upload their data to be scored with EAP scoring methods. An alternative to the HealthMeasures Scoring Service is a “look-up” table to convert a raw sum score to an EAP score. The scores in these look-up tables are calculated with EAP methods and represent the most probable theta level across all possible response pattern combinations for a single scale-level sum score [14, 28]. The maximum and minimum scale-level scores in the table relate to the floor and ceiling of the scale. Table 2 shows an example look-up table.

**Table 2:** IRT to Raw Sum Score Look-up Table

Raw Sum score	IRT Theta score
3	− 3.59
4	− 3.36
5	− 3.15
6	− 2.96
7	− 2.78
8	− 2.60
9	− 2.42
10	− 2.23
11	− 2.03
12	− 1.81
13	− 1.54
14	− 1.18
15	0.21

**Fig. 9:**
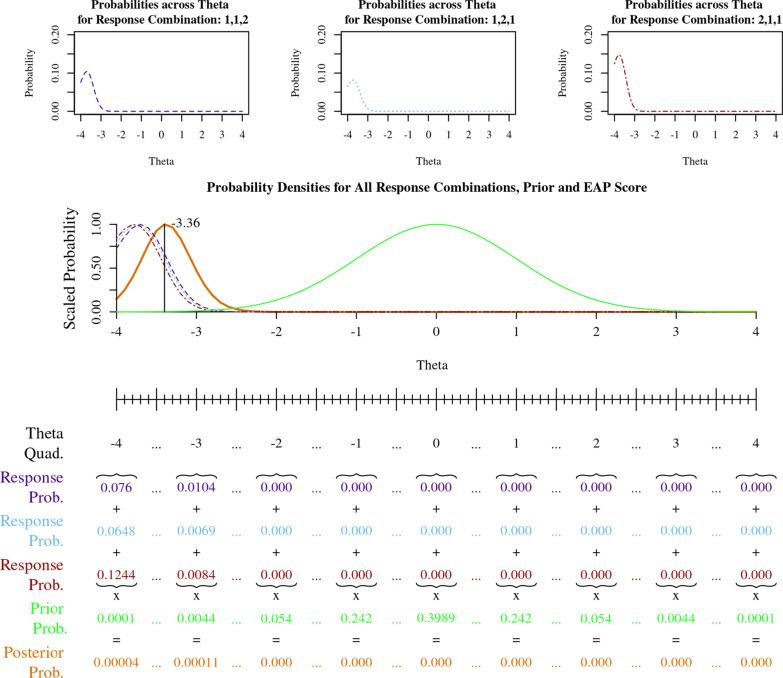
Calculation of most likely IRT score for raw sum score of 4

Figure 9 shows an example of how an EAP score for a raw sum score of 4 in Table 2 is calculated. In this example, three Physical Function items (PFA51, PFB25 and PFC46) make up a three-item scale. The minimum possible scale score on the three item scale is 3 (all three items have a raw score of 1) and maximum scale score of 15 (all three items have a raw score of 5), as shown in Table 2.

To calculate the EAP score for a scale-level raw sum score of 4, we first calculate the theta probabilities for each of the three possible combinations that sum to 4. Each response combination includes two 1’s and one 2, i.e., 1,1,2; 1,2,1; 2,1,1. Each of the response probability curves are shown in the top three plots of Fig. 9. The total probability of multiple independent events (or in this case, three independent response patterns which each have a sum-score of 4) can be found by summation, shown in bottom of Fig. 9. The center plot in Fig. 9 shows each scaled probability curves, including the three dotted, dashed and dot-dashed response pattern probability curves, and their sum multiplied by the prior. The result is a posterior probability curve (bold solid orange line) with a theta maximum likelihood of − 3.36 for all response combinations which sum to 4.

**Fig. 10:**
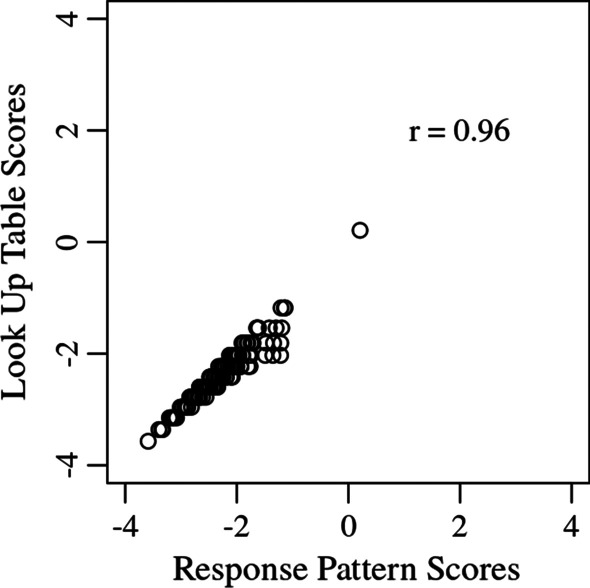
Comparison of look-up and pattern response Expected A Posteriori scoring methods

In order to differentiate between the two forms of scoring, one is referred to as “response pattern scoring” or “pattern response scoring” because it uses an individual’s pattern of responses and the other is referred to as “look-up table scoring.” Scores calculated for a look-up table are typically very highly correlated (e.g., > 0.9) with response pattern scoring. Figure 10 shows a plot of look-up and pattern response scoring methods for all response option combinations of the three physical function items used in Table 2 and Fig. 9. The two scoring methods have a pearson correlation coefficient of 0.96.

It is important to recognize that relative ease of use of look-up tables is balanced by a loss in resolution in comparison to pattern response scoring. Look-up scoring treats responses of equal raw score values (1, “Unable to do”) as equal, even if the responses relate to items of unequal difficulty (“Are you able to go for a walk of at least 15 min?” and “Are you able to run or jog for two miles (3 km)?”). This results in score differences or error between the pattern-response scoring and look-up table methods. The choice of implementing pattern response scoring or look-up scoring should reflect the context of measurement (e.g., regulatory decision making) and the corresponding level of precision needed. Pattern response scoring methods are more sensitive to an individual’s pattern of responses and are recommended whenever possible, and where appropriate, look-up table scoring is a good alternative.

### Posterior standard deviation and standard error

Because of the inclusion of the prior in estimating the theta score, EAP scores don’t have a traditional standard error. Instead, we can calculate the standard deviation of the posterior distribution. The method for calculating the posterior standard deviation is the same for both pattern response and look-up table scoring methods. Formula (4) details the calculation of the posterior standard deviation.

There are parallels between the posterior standard deviation and the common standard deviation formula (5), notably, the size of the numerator of both formulas is driven by the sum of squared deviations from a single point, either the EAP score in Formula (4) or the mean in Formula (5) and both Formulas use a square root. They differ in that the squared deviation at each level of the theta quadrature is multiplied by the posterior probability before summation in Formula (4), and that the sum of the posterior distribution is the denominator in Formula (4) and the sample size is in the denominator of Formula (5).

4 $$Posterior\;\;SD = \sqrt {\frac{{\sum \left( {Posterior*\left( {Theta\;\;Quadrature - EAP\;\;Score } \right)}^{2} \right)}}{{\sum \left( {Posterior} \right)}}}$$

5 $$SD = \sqrt {\frac{{\sum \left( {X - \overline{X}} \right)^{2} }}{N}}$$

While the posterior standard deviation is not a standard error, it is related in a number of ways. The posterior standard deviation is a function of the shape of the posterior probability curve, which is informed by the consistency of response probabilities and the number of items scored.

**Fig. 11:**
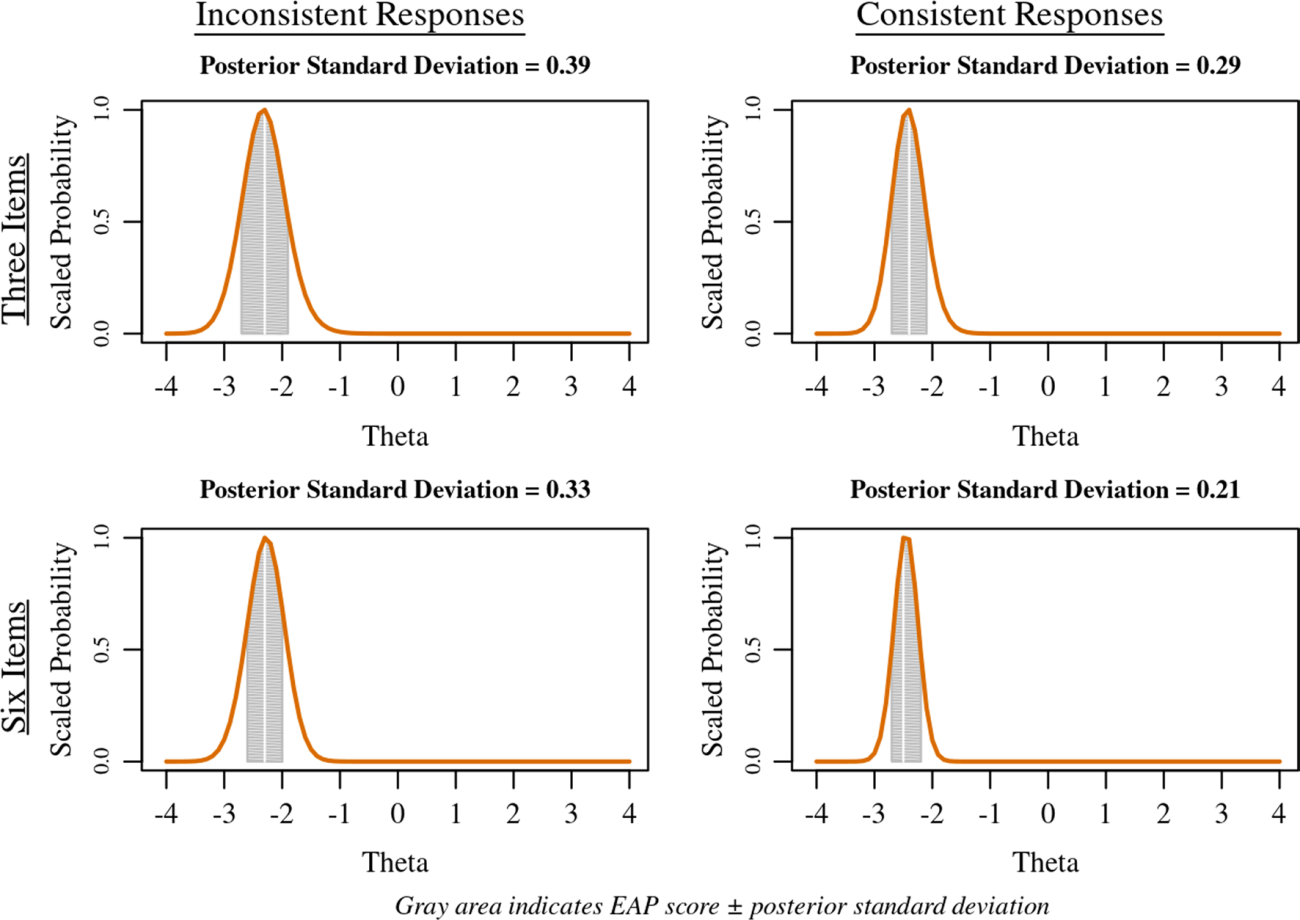
Posterior standard deviation, number of items and response consistency

Figure 11 shows an example of the relationship between the number of items scored (e.g., 3 or 6 items), consistency of item responses (e.g., raw scores of 3,3,3 or 1,3,5) and the resulting posterior standard deviation. The gray shaded area under the bold solid orange posterior probability curve in Fig. 11 indicates a bandwidth of one standard deviation from the EAP score. Generally, a smaller posterior standard deviation occurs with a larger number of items with consistent responses, which maps onto a smaller standard error. Conversely, a smaller number of inconsistent item responses leads to a larger posterior standard deviation and larger standard error. Bock draws a direct and “near identity” relationship between the posterior standard deviation and standard error as the number of items increases (p. 437) [15].

Similar to how T-scores are a linear transformation of theta (Formula (3)), posterior standard deviations can be put on the T-score metric by multiplication by 10, e.g., a posterior standard deviation of 0.21 on the theta metric is a posterior standard deviation of 2.1 on the T-score metric.

## Conclusion

Expected A Posteriori (EAP) scoring is a flexible and efficient scoring method that can be visualized and logically explained. Item response option probabilities distributed across a latent trait spectrum, theta, are the building blocks of EAP scoring and the maximum likelihood of these probabilities can provide a score estimate. An EAP score represents the level of latent trait experienced by the respondent compared to the level of latent trait present in the people who make up the calibration sample. Introduction of a theta quadrature and a Bayesian “prior” simplifies complex mathematical operations and alleviates measurement problems. For users who don’t have access to the statistical code and item calibration statistics, a scale-level raw sum score to EAP score look-up table can be calculated for custom short-forms or accessed on HealthMeasures website for existing short-forms. A posterior standard deviation can be calculated for all EAP scoring methods, which reflects the score standard error. A more complete understanding of the operation and options in PROMIS EAP scoring will help ground PROMIS IRT methods with existing users and will support the further adoption and implementation of PROMIS among researchers, clinicians, industry sponsors and regulators.

## Data Availability

All materials used in this manuscript are openly available and included in the parent R Markdown document, which will be submitted with the manuscript. Additionally, statistical code for performing Expected A Posteriori scoring is provided in the appendices of the manuscript. No data was used in the creation of this manuscript.

## Appendix

### ThetaSEeap.R

The “ThetaSEeap.R” script is an R script for calculating “pattern response” EAP scores, and was originally written by Choi [29].

### RSSS.R

The “RSSS.R” script is an R script for calculating EAP to raw sum score “Look Up” tables, and was originally written by Choi [30].

## Abbreviations

CAT: Computer adaptive test
EAP: Expected A Posteriori
IRT: Item response theory
PROMIS: Patient reported outcomes measurement information system
SD: Standard deviation
